# Factors influencing spinal curvature measurements on ultrasound images for children with adolescent idiopathic scoliosis (AIS)

**DOI:** 10.1371/journal.pone.0198792

**Published:** 2018-06-18

**Authors:** Rui Zheng, Doug Hill, Douglas Hedden, James Mahood, Marc Moreau, Sarah Southon, Edmond Lou

**Affiliations:** 1 School of Information Science and Technology, ShanghaiTech University, Shanghai, China; 2 Department of Surgery, University of Alberta, Edmonton, Alberta, Canada; 3 Department of Electrical and Computer Engineering, University of Alberta, Edmonton, Alberta, Canada; Medical College of Wisconsin, UNITED STATES

## Abstract

The measurements of spinal curvatures using the ultrasound (US) imaging method on children with scoliosis have been comparable with radiography. However, factors influencing the reliability and accuracy of US measurement have not been studied. The purpose of this study is to investigate the effects of curve features and patients’ demographics on US measurements and to determine which factors influence the reliability and accuracy. Two hundred children with scoliosis were recruited and scanned with US by one experienced operator and three trainees. One experienced rater measured the proxy Cobb angles from US images twice one week apart and compared the results with clinical radiographic records. The correlation and accuracy between the US and radiographic measurements were subdivided by different curve severities, curve types, subjects’ weight status and US acquisition experiences. A total of 326 and 313 curves were recognized from radiographs and US images, respectively. The mean Cobb angles of the 13 missing curves were 17.4±7.4° and 11 at the thoracic region. Among the 16 curves showing large discrepancy (≥6°) between US and radiographic measurements, 7 were main thoracic and 6 were lumbar curves. Twelve had axial vertebral rotation (AVR) greater than 8°. The US scans performed by the experienced operator showed fewer large discrepancy curves, smaller difference and higher correlation than the scans from the trainees (3%, 1.7±1.5°, 0.95 vs 6%, 2.4±1.8°, 0.90). Only 4% missing and 5% large discrepancy curves were demonstrated for US measurements in comparison to radiography. The missing curves were mainly caused by small severity and in the upper spinal region. There was a higher chance of the large discrepancy curves in the main thoracic and lumbar regions with AVR>8°. A skilled operator acquired better US images and led to more accurate measurements especially for those subjects with larger curvatures, AVR and body mass index (BMI).

## Introduction

Adolescent idiopathic scoliosis (AIS) is a three-dimensional deformity of the spine that presents with lateral curvature and vertebral rotation. The standard clinical practice to diagnose and monitor AIS is to measure the Cobb angle on a standing posteroanterior (PA) radiograph. The Cobb angle is the gold standard to determine the severity of the spinal curvatures [1]. However, taking radiographs exposes patients to ionizing radiation which is always a concern. Recently the EOS system featured with low dose biplanar radiograph has been introduced to obtain the anteroposterior and lateral images of scoliotic subjects [2]. Although EOS system is becoming more common, the cost of the EOS system is too high, and low income countries and many private scoliosis clinics still use standard x-ray machines to acquire radiographs. Furthermore, the EOS system also requires significant amount of spacing and installation cost. On the other hand, the cumulative radiation exposure of these radiographs is still associated with increased cancer risk. In 1989, Suzuki et al. reported an ultrasound (US) imaging technique, which did not expose patients to ionizing radiation and could estimate the curvature of the spine [3]. They demonstrated that US could be used to outline the spinous processes and laminae. By combining these 2 features and the information about angles obtained using a protractor, they were able to estimate the axial vertebral rotation (AVR). They found a strong linear correlation between the AVR and Cobb angle in untreated patients. Burwell et al. applied real-time US imaging to measure rib rotation in prone positions and compared the results with the measurements of apical AVR. They reported that the mean apical spine-minus-rib rotation difference (SRRD) was 7°, and the spinal rotation was always greater than the rib rotation [4,5].

Recently a new US imaging method [6–19] based on combining continuous ultrasound scanned frames (B-mode images) to reconstruct a full spine image has been introduced to directly measure the spinal curvature instead of inferring the Cobb angle from the AVR. The reconstructed spinal image was three-dimensional (3D) and could be projected onto a two-dimensional (2D) plane so that the traditional Cobb measurement protocol could be used to measure the proxy Cobb angles on US images. A free hand 3D US imaging system developed by Cheung et al [6,7] was validated on a spine phantom with 16 simulated deformity configurations. A strong correlation (R^2^ = 0.76) was obtained between the radiographic and ultrasound measurements, and the ICC value of intra- and inter-rater correlations were 0.99 and 0.89 respectively. Chen et al. [8,9] also conducted *in-vitro* studies on cadaver spinal phantoms to determine better landmarks to be used for the measurement of the spinal curvatures. They scanned 30 scoliotic configurations of varying spinal deformities using both the US and laser scanner (LS) systems. They showed the center of lamina (COL) method applied on the US images provided highly reliable measurement results to assess the proxy Cobb angles. There were no significant differences between the Cobb values measured from the LS images versus those from the US images. The mean absolute difference (MAD) between the two measurements was <4°. Another *in-vitro* study was performed by Ungi et al.[10]. The transverse processes on the US images were used as vertebral landmarks to estimate the spinal curvatures. Their method was tested on both adult and pediatric spine phantoms, and the MAD and the standard deviation (SD) between the ultrasound (the transverse processes method) and radiographic measurements (the Cobb method) were 1.27 ± 0.84° and 0.96 ± 0.87°, respectively. For the AVR assessment, Vo et al. [11] scanned three cadaveric vertebrae T7, L1, and L3 setting the vertebral rotation from 0 to 40° with 5° increments for each vertebra. They measured the AVR using the COL method, and the MAD±SD values between the actual values and the average US measurements of three raters were 1.5±0.3°, 1.6±0.3°, and 1.7±0.5°, respectively.

The US method has also been applied to the *in-vivo* studies to compare with the measurements from PA radiographs and MRI images. A pilot clinical study using the COL method to measure proxy Cobb angles was performed on 26 AIS subjects by Zheng et al [12]. In that study, the ICC[2,1] values of the intra- and inter-rater reliability of the proxy Cobb angle were all greater than 0.80. The US and radiographic measurements showed a good agreement with correlation coefficient (R) 0.78–0.84 for 3 raters and the average standard error of measurement (SEM) was 3.1°. The aid of a previous radiograph (AOR) method was then developed to improve the accuracy and reliability of US measurements [13,14]. By overlaying the current US image on top of the previous radiograph, observers can interpret the US image more accurately. The correlation between the US and radiographic measurements was 0.90 and the MAD was 2.8° for the AOR method, and it showed significant improvement in comparison to the results of the blinded US method, 0.73 and 4.8° respectively. Wang et al. [15] presented the comparison results of lateral curvature measurements between the US and MRI images. All patients were lying in supine position and a specific bed was designed to allow the US to be scanned underneath the bed. In this *in-vivo* study, thirty scoliotic curves (Cobb angle range 10.2°–68.2°) from sixteen AIS patients were identified. The US and MRI methods showed no significant difference (p<0.05) on the lateral curvature measurement. They also demonstrated a good agreement using the Bland-Altman method and a high Pearson correlation coefficient (R>0.9, p<0.05). Zheng et al. [16] and Cheung et al. [17] applied a radiation-free freehand 3-D ultrasound system using a volume projection imaging method to investigate the intra- and inter-reliability between operators and raters. The ICC values of intra-rater and intra-operator test for Scolioscan angle measurement were larger than 0.94 and 0.88 respectively, and the ICC values of inter-rater and inter-operator test were both larger than 0.87 [16]. On a study involving 36 scoliotic subjects, the spinal curvature obtained by the ultrasound imaging method showed good linear correlations with the radiographic Cobb method (R^2^ = 0.8, P<0.001) [17]. In the most recent study on AVR evaluation, Chen et al. [18] selected 48 vertebrae from 18 spine curves and measured the AVR using the COL method on the US transverse images and the Stokes’ method on radiographs. The US COL method presented good intra- and inter-observer reliability with ICCs>0.91 and MADs<1.4°. The US and radiographic measurements showed the MADs of 2.7–3.5° for the AVR assessment. Wang et al. [19] performed the study on measuring the apical vertebral rotation using the US COL method and the MRI Aaro-Dahlborn method. The intra- and inter-reliability of the US measurements was very reliable (both ICC[2, K]>0.9, p< 0.05). The US and MRI results showed no significant difference (p< 0.05), and the high correlation demonstrated by the Bland-Altman method was found (r>0.9, p<0.05).

Even though much research had reported the reliability and accuracy of lateral curvatures and AVR on using ultrasound imaging techniques for children with AIS, a large clinical validation and factors which influence the reliability and accuracy of US measurements have not been analyzed. Therefore, the objectives of this study are to investigate the effects on the US measurements caused by different curve characteristics such as curve severity, curve type, subjects’ weight status and US acquisition experiences.

## Methods

### Subjects

A total of 200 adolescents who had scoliosis (F:170, M:30, Age: 14.6±1.9) were recruited from the local scoliosis clinic between September 2013 and April 2015. The inclusion criteria were patients who: 1) were diagnosed with JIS or AIS; 2) had no prior surgical treatment; 3) had out-of-brace radiographs on the study day; 4) had at least one previous out-of-brace PA radiograph, and 5) the major Cobb angle from the previous radiographic measurement was between 10° and 45° (mild (10° to 24°) and moderate (25° to 45°)). Ethics approval was granted by the University of Alberta Health Ethics Research Board. Subjects who were under 14 years old signed their written assents and their guardians signed the parental consents, while those who were over 14 years old signed their own written consents before being enrolled into the study.

### Data acquisition and measurement

**Fig 1:**
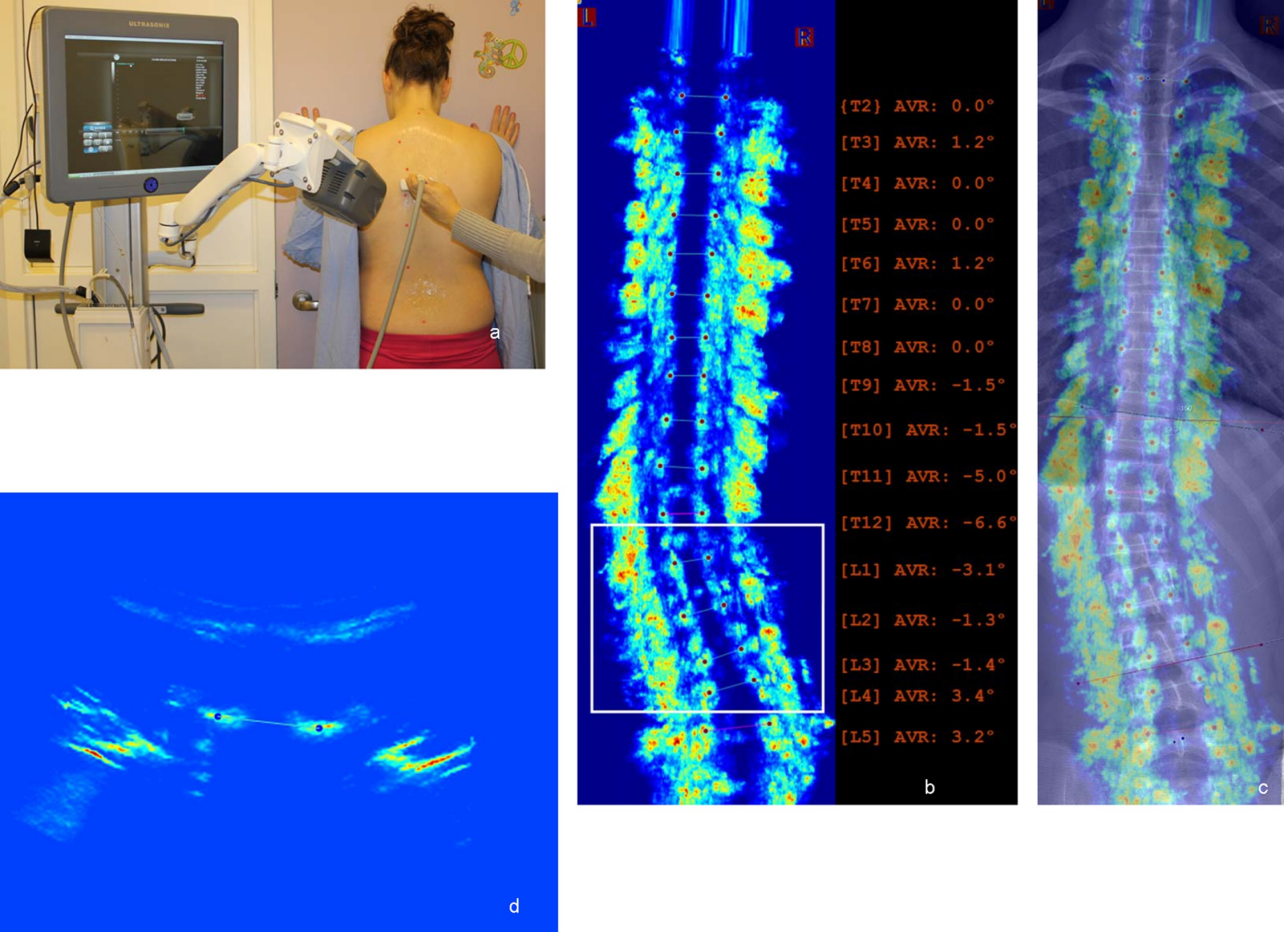
The US measurement on a scoliotic subject. (a) the subject being scanned using SonixTABLET medical ultrasound system, (b) the US coronal image with the measurements of vertebral rotation, (c) the overlaid US coronal image on the previous radiograph, and (d) the US transverse image at apical level (T12).

The free standing ultrasound scan and a PA radiograph were obtained on each subject within one hour on the study day. The SonixTABLET medical ultrasound system equipped with GPS transmitter and transducer (Analogic Ultrasound—BK Medical, Peabody, MA) were used to acquire the US scan, and the scan followed the standard procedure described in [12,14]. As shown in Fig 1A, the patient’s hands were put against the wall and the arms were holding on the chest level to preventing the body leaning back and forth. This posture was similar to the one used during radiography. A transducer was pushed against the subject’s back and moved downward from C7 to L5 along the curve of the spine in a standing position. It took less than 1 minute to acquire the entire spine data. Custom in-house software was developed to reconstruct and measure the 3D image data. The reconstruction and measurement time were approximately 8 minutes. During this long enrollment period for data acquisition, 4 ultrasound operators were involved to scan subjects. The first 3 operators were student trainees who scanned the first 120 recruited subjects, and the remaining 80 subjects were scanned by the last operator, an experienced ultrasound researcher who had one-year experience on ultrasound image acquisition and scanned more than 100 subjects.

One rater who had 3-year experience on proxy Cobb angle assessment on ultrasound images measured all 200 images twice one week apart. The center of lamina (COL) [8,9] and aid of previous standing radiographs (AOR) [13,14] methods were applied for this study and the rater was blinded to the radiographic measurements. To implement the AOR method, the most recent radiograph (out of brace) prior to this study was exported on each patient. The average time duration between the previous radiograph and the study day was 8.7±3.5 months. The AVR was measured on the US transverse images of the apical and its superior and inferior vertebrae on each curve using the method from Vo et al [11]. The maximum AVR among these three levels was recorded and referred as the AVR of the curve for further analysis. The custom developed software was applied for the measurements on both the US images and the previous radiographs. Fig 1B showed the US coronal image, Fig 1C showed the overlapped image between the US coronal image and the corresponding previous radiograph and Fig 1D showed the transverse images of a single vertebra. After aligning the lamina line which indicated the vertebral level on US images with the vertebra on the radiograph, the software automatically calculated and displayed the lateral curvatures and axial vertebral rotation of each level as illustrated in Fig 1B.

The clinical records of the Cobb angles measured from the radiographs acquired on the study day were blinded from the rater, exported from the local scoliosis database after the rater completed the ultrasound measurements and used as the reference to assess the accuracy of the US measurements of the lateral curvatures.

### Statistical analysis

The accuracy of the spinal curvature measurement was evaluated by comparing the results of this study with a previous study [14] with the same rater, which included the mean absolute difference (MAD), the standard deviation (SD), the standard errors of measurement (SEM), the intraclass correlation coefficients using a two-way random model and absolute agreement with confidence interval of 95% (ICC[2,1]) and the error index of vertebral level selection (EI) [9,14,20]. The MAD, SD and the coefficient of determination (*R*^*2*^) were computed to assess the difference and correlation between the US and radiographic measurements. The Bland-Altman plot was used to investigate the agreement between the two methods, and the two lines indicating Mean±1.96SD represented the limits of agreement [21]. The statistical analysis was performed using the IBM SPSS Statistics version 21 and Microsoft Excel 2010. Based on Currier’s characterization [22], The ICC value was found to be very reliable (0.80–1.00), moderate reliable (0.60–0.79) and questionable reliable (≤0.60).

All the curves were categorized into two groups according to the Cobb angle (curve severity) either <25° (mild curve) or greater than 25° (moderate curve). The different curve locations were defined according to the locations of the apical vertebrae as upper thoracic curve (T2-T6), main thoracic curve (T7-T11), thoracolumbar curve (T12-L1) and lumbar curve (L2-L4) [23].

All patients were also divided into different weight status groups based on gender, age and body mass index (BMI) in reference to the BMI-for-age percentile growth charts [24]. The weight status was categorized as underweight (<5%), normal or healthy (5%-85%) and overweight (≥85%). If there was a lack of weight and height information in the database, the patients were denoted as not applicable (N/A).

Two kinds of curve were specifically defined and analyzed. The missing curve was the curve which was measured on the radiograph but not detected by the US measurements. The large discrepancy curve was the curve whose measurement difference between US and radiographic measurements was greater than the clinical acceptable error (5°) [1].

## Results

Table 1 lists the curve information and measurements in comparison to a previous study [14]. The curve information was summarized and calculated based on the radiographic measurement which was extracted from the local scoliosis database. The results showed consistency with the previous research. The intra-rater reliability maintained the same level with the ICC value 0.95 and SEM 1.7°. The MAD and R^2^ between the US and radiographic measurements were improved from 2.7° and 0.87 to 2.1° and 0.92, respectively.

**Table 1:** The curve information and the comparison of measurements between this study and the previous study[14].

		This study	Previous study[14]
Curve No		326	109
Current Curve range		10–53°	10–46°
Mean ± SD		23.7 ± 9.5°	24.8 ± 9.0°
No. of Curves at different locations	Upper Thoracic (UT)	30 (9%)	6 (6%)
	Main Thoracic (MT)	138 (42%)	45 (41%)
	Thoracolumbar (TL)	63 (19%)	27 (25%)
	Lumbar (L)	95 (29%)	31 (28%)
Intra-rater reliability	MAD ± SD	2.0 ± 1.7°	1.8 ± 1.5°
	ICC[2,1]	0.96	0.95
	SEM	1.7	1.7
US vs Radiography	MAD ± SD	2.1 ± 1.7°	2.7 ± 1.9°
	R^2^	0.92	0.87
	EI	1.1 ± 1.1	0.9 ± 1.0

The missing curves and large discrepancy curves in different curve severities, curve locations, weight status and US acquisition experience were presented and compared in Table 2. Due to the limited range of Cobb angle (10–24°) for the mild curves, the correlation *R*^*2*^ between the US and radiographic measurements in this category was only 0.64, however the MAD and SD remained similar to the moderate curve measurements.

**Table 2:** The US measurement results in different curve severities, curve locations, subjects’ weight status and US acquisition experiences.

		Number of Curve			MAD (°)	SD (°)	*R*^*2*^
		Total	Missing Curve*	Large discrepancy*			
Overall		326	13 (4%)	16 (5%)	2.1	1.7	0.92
Curve severity	<25^o^	188	**11 (6%)**	6 (3%)	2.1	1.6	0.64
	≥25^o^	138	2 (1%)	**10 (7%)**	2.2	1.9	0.85
Curve location	Upper Thoracic (UT)	30	**5 (17%)**	1 (3%)	1.9	1.4	0.90
	Main Thoracic (MT)	138	**6 (4%)**	**7 (5%)**	2.2	1.8	0.94
	Thoracolumbar (TL)	63	1 (2%)	2 (3%)	2.0	1.6	0.90
	Lumbar (L)	95	1 (1%)	**6 (6%)**	2.3	1.9	0.90
Weight status	Normal	235	8 (3%)	10 (4%)	2.2	1.7	0.92
	Overweight	63	3 (5%)	3 (5%)	2.1	1.7	0.92
	Underweight	16	1 (6%)	0	1.8	1.5	0.85
	N/A	12	1 (8%)	1 (8%)	2.6	2.0	0.92
Acquisition experience	Trainee	200	9(5%)	12 (6%)	2.4	1.8	0.90
	Experienced	126	4 (3%)	4 (3%)	1.7	1.5	0.95

* The ratio in brackets is the number of missing or large discrepancy curves divided by the total number of curves on that category

Among the 13 missing curves, 11 were in mild severity range and 2 were moderate curves. The mean Cobb angle of all missing curves was 17.4±7.4° which was significantly smaller (*p*<0.01) than the mean Cobb angle of all curves 23.7 ± 9.5°. The two missing moderate curves were at the upper thoracic region with Cobb angles 25° and 38°. Furthermore, 85% of the missing curves (11 out of 13) were in either the upper thoracic or main thoracic regions. Among the 11 missing thoracic curves, 5 of those had the upper vertebral levels at T1 and T2. The two missing thoracolumbar and lumbar curves their Cobb angles were 12° and 14°, respectively.

Sixteen curves in 12 subjects showed a large discrepancy (≥6°) between the US and radiographic measurements. The maximum difference was 9° and the MAD±SD of all 16 large discrepancy curves was 6.7±0.9°. The ≥25^o^ curve group showed a higher percentage of large discrepancy curves (7%) than the mild curve group (3%).

Table 3 shows the curvature measurement in different AVR groups. In this study, the AVR was measured directly from the US transverse images and the average of the 313 measurements from curves on the US was 7.8±4.3°. Therefore, 8° was chosen as the threshold to differentiate the small and large apical AVR groups. The large apical AVR more often occurred in the case of large curve severities, and the mean Cobb of the large AVR group was 9° greater than the small AVR group. A large discrepancy occurred more frequently in the large apical AVR cases, 75% versus 25%. For the different curve locations, the large discrepancy curves mainly occurred on the main thoracic and lumbar regions (7 and 6, respectively). The ultrasound data acquired from the experienced operator showed fewer large discrepancy curve measurements than the trainee operators (3% vs 6%).

**Table 3:** The US measurement results in the different AVR groups.

AVR	Number of Curve		Cobb Mean (°)	Cobb SD (°)	MAD (°)	SD (°)	*R*^*2*^
	Total	Large discrepancy*					
Overall	313	16 (5%)	24.0	9.5	2.2	1.7	0.92
≤8°	189	4 (2%)	20.4	7.7	2.1	1.5	0.89
>8°	124	12 (10%)	29.5	9.5	2.2	2.0	0.91

* The ratio in brackets is the number of large discrepancy curves divided by the total number of curves on that category.

**Fig 2:**
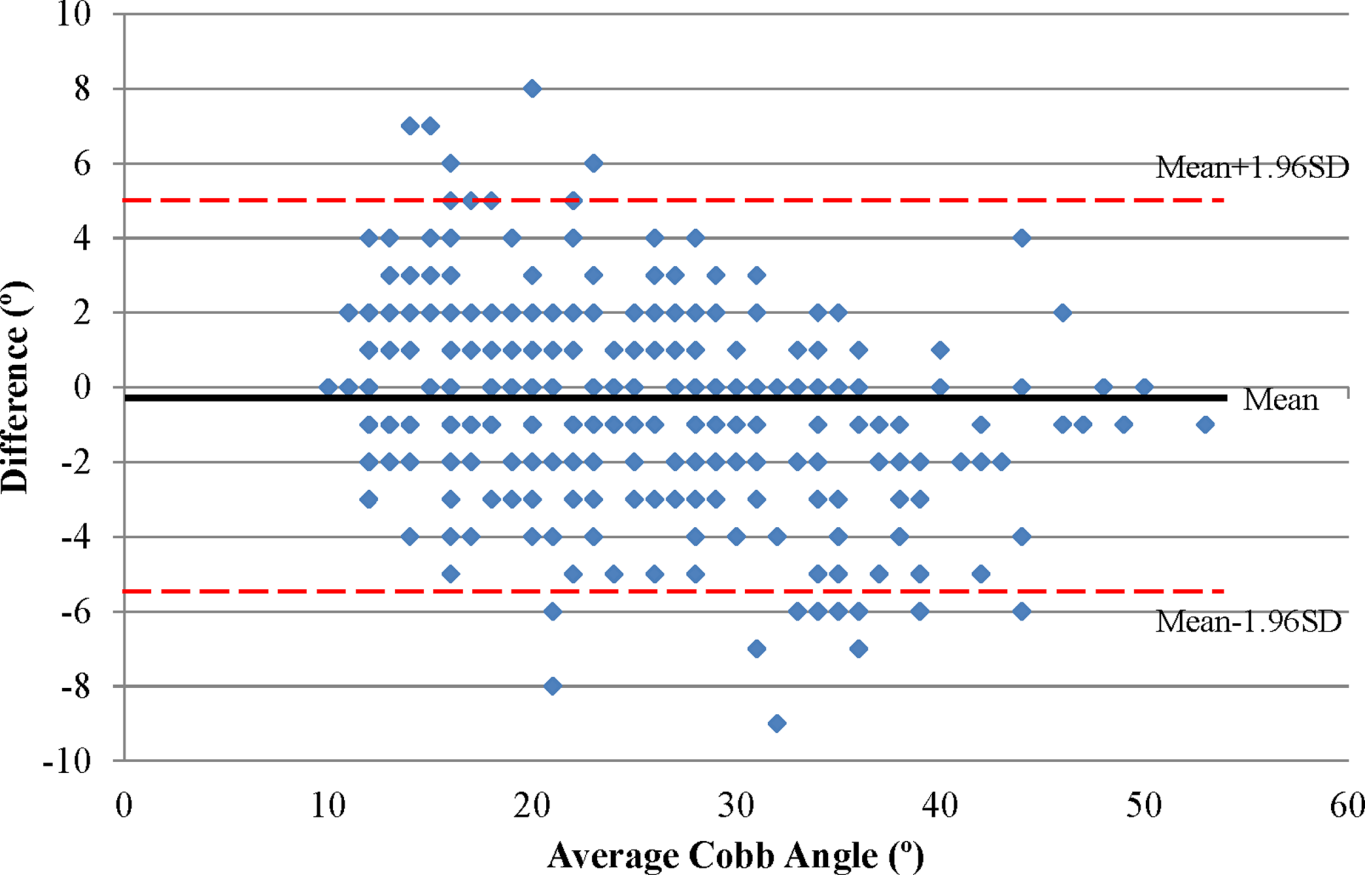
The comparison between radiographic and US measurement on Cobb angles. a) the regression line between radiographic and US proxy Cobb angles, b) Bland-Altman plot between the measurement difference on the US proxy Cobb minus the radiographic Cobb angle versus the average Cobb angles between US and radiographic measurements.

Fig 2 illustrates the comparison between radiographic and US measurements on the curvatures. The US and radiographic measurements showed high correlation coefficient (R^2^ = 0.92) (Fig 2A), and the Bland-Altman method demonstrated good agreement between the two methods as shown in Fig 2B. The mean measurement difference on the US proxy Cobb minus the radiographic Cobb angle was -0.3°, and the 95% limits of agreement were -5.6°-5.0°. There were 16 data points were out of the limit range which were also large discrepancy curves, and 5 points showed positive large discrepancy (>5°) and 11 points showed negative large discrepancy (<-5°).

The BMI varied from 14.3 to 37.3 in the age range of 10.2–18.3 for 200 subjects. However, it indicated no apparent difference on the measurement accuracy for the normal and overweight status (2.2±1.7° vs 2.1±1.7°) and a slightly smaller MAD value for the underweight status (1.8±1.5°) as shown in Table 2.

## Discussion

In this study, the same rater who measured the ultrasound images in the (Zheng, 2016) study [14] obtained the equivalently good accuracy and reliability results. There were slight improvements on the MAD and correlations between US and radiographic measurements comparing this study to the previous study (2.1±1.7°/92% vs 2.7±1.9°/87%, respectively). These improvements might be partially due to gaining measurement experience.

The ratio of missing curves for the US measurements on all curves was 4%. The two most common scenarios were mild severity of the curves and thoracic curve locations. Even though the ultrasound operator guided the candidates to stand in a standard posture during the ultrasound scan, it was inevitable that the posture during the US scan might not exactly match that of the radiograph. The strong pressure applied to maintain good contact between the transducer and the subject’s back could also affect the standing posture during the ultrasound scan. Therefore, the curve magnitude could be influenced by the change of the standing postures and result in undiscovered curves. A custom designed frame which can standardize the position of the candidate’s upper body and prevent the torso from unexpected leaning, tilting and rotating, may help to minimize the change of the subject’s posture caused by the different standing positions. In addition, since the transducer is in a convex shape and the contact area at the C7 to T2 region is relatively small, there is a high possibility that the image quality in that region is poor. This notably affected the recognition and determination of end vertebra for the curves with end vertebra at either the T1 or T2 level. This can cause the failure of detecting these curves.

There were 5% of the overall curves that the measurement difference between the US proxy Cobb and the radiographic Cobb angles was greater than the clinical acceptable error (5°) [1]. Even though the large discrepancy curves were a very small portion of all curves, there were several features observed during the analysis. The two main influencing factors may be the large AVR (≥8°) occurring with the larger curve severities and the curve locations, especially in the main thoracic and lumbar regions. Both of these affect the appearance of the vertebral structures on the US images and increase the difficulty of detecting and identifying the center of the lamina.

**Fig 3:**
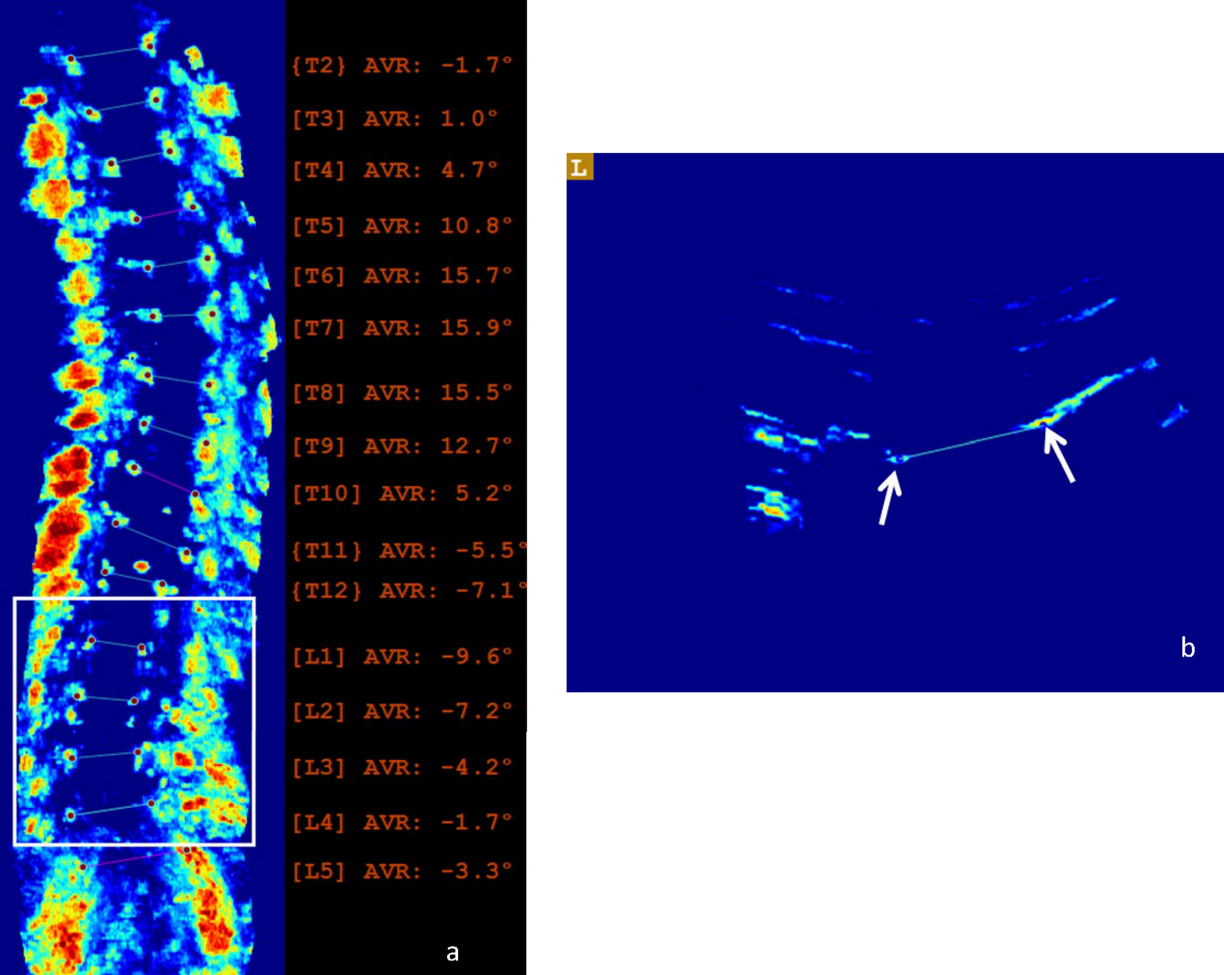
The US images from a patient with the maximum AVR of 16°. (a) the coronal image, and (b) the transverse image.

**Fig 4:**
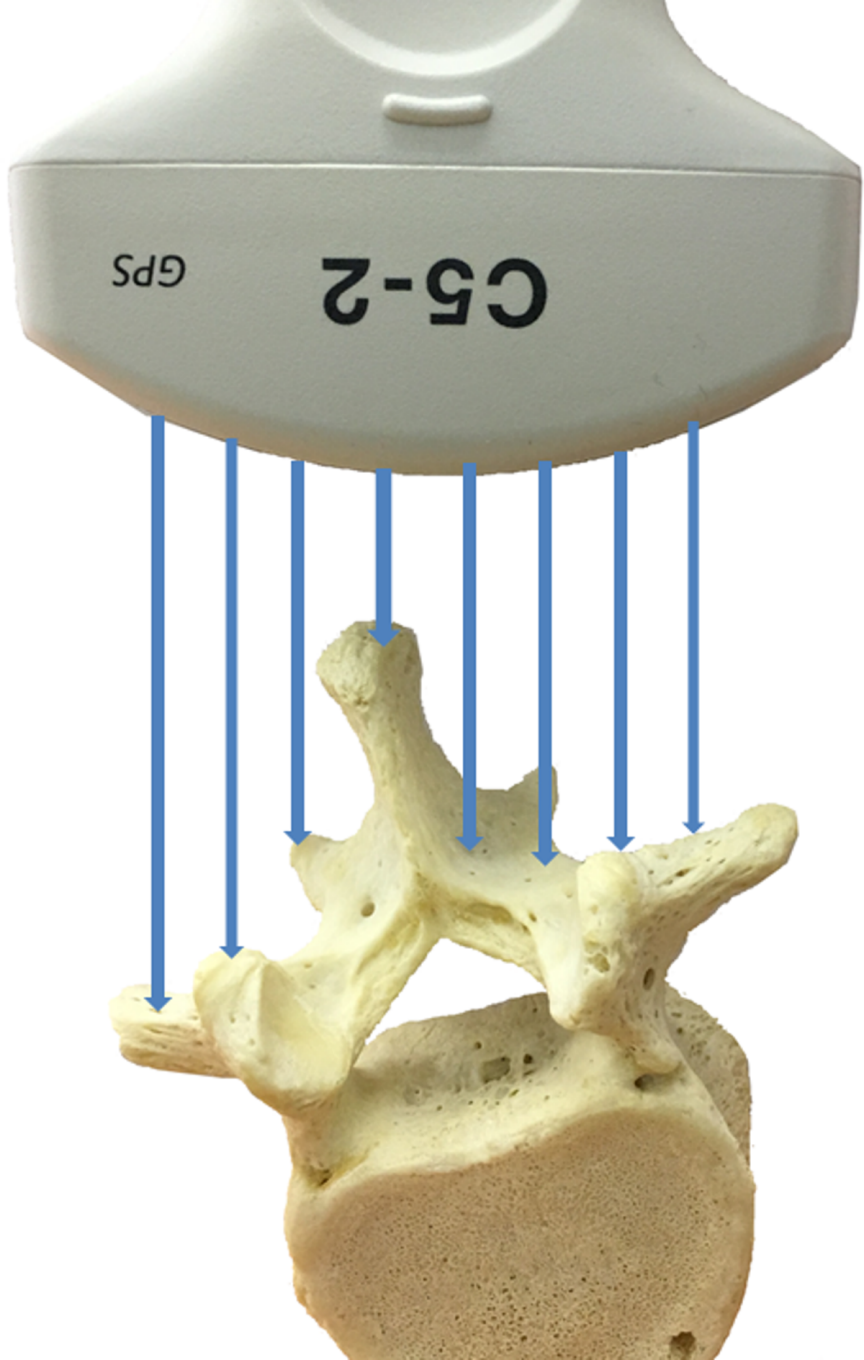
The schematic of ultrasound beam propagating from the transducer to a tilted vertebra.

Fig 3A shows the US coronal image and Fig 3B shows the transverse image from a patient with a maximum AVR of 16°. As indicated by the arrows in Fig 3B, the right side of the lamina can be easily identified as a long bright line; however, the image intensity on the left side is much lower and the lamina is only shown as a short weak line. This display difference mainly is due to the unequal reflection energies from the lamina areas which are caused by the vertebral rotation. As illustrated in Fig 4, since the tilted spinous process interfered with the ultrasound beam, the right lamina reflected more energy back to the transducer than the left one. Consequently, the lamina on the coronal image (white box in Fig 3A) was incomplete and showed lower contrast and brightness compared to the ordinary lamina region (white box in Fig 1B). A complete image of the lamina can help to accurately detect the slope of the vertebra which provides better measurement results. The large AVR normally occurred in the curves with larger Cobb angles; as a result, it presented a higher percentile of the large discrepancy curves with a Cobb angle ≥25° than the mild curves (7% vs 3%).

The accuracy of the US measurements was also influenced by curve location; the large discrepancy curves were mostly main thoracic and lumbar curves. In the main thoracic region, especially for the subjects with larger vertebral rotation, the spinal curve will result in rotation of the rib cage which causes one side to protrude out on the subject’s back. The uneven back surfaces in conjunction with the deeper posterior median furrow in the main thoracic area create gaps between the transducer and the scanning region in the middle back of the subject. This poor contact can cause ultrasound signal loss or reduction and hence generate a poor US image. In addition, the rotated ribs may be located in between the transducer and vertebra; the reflection energy from the lamina could be partly blocked or redirected, and the image quality can be compromised as well.

**Fig 5:**
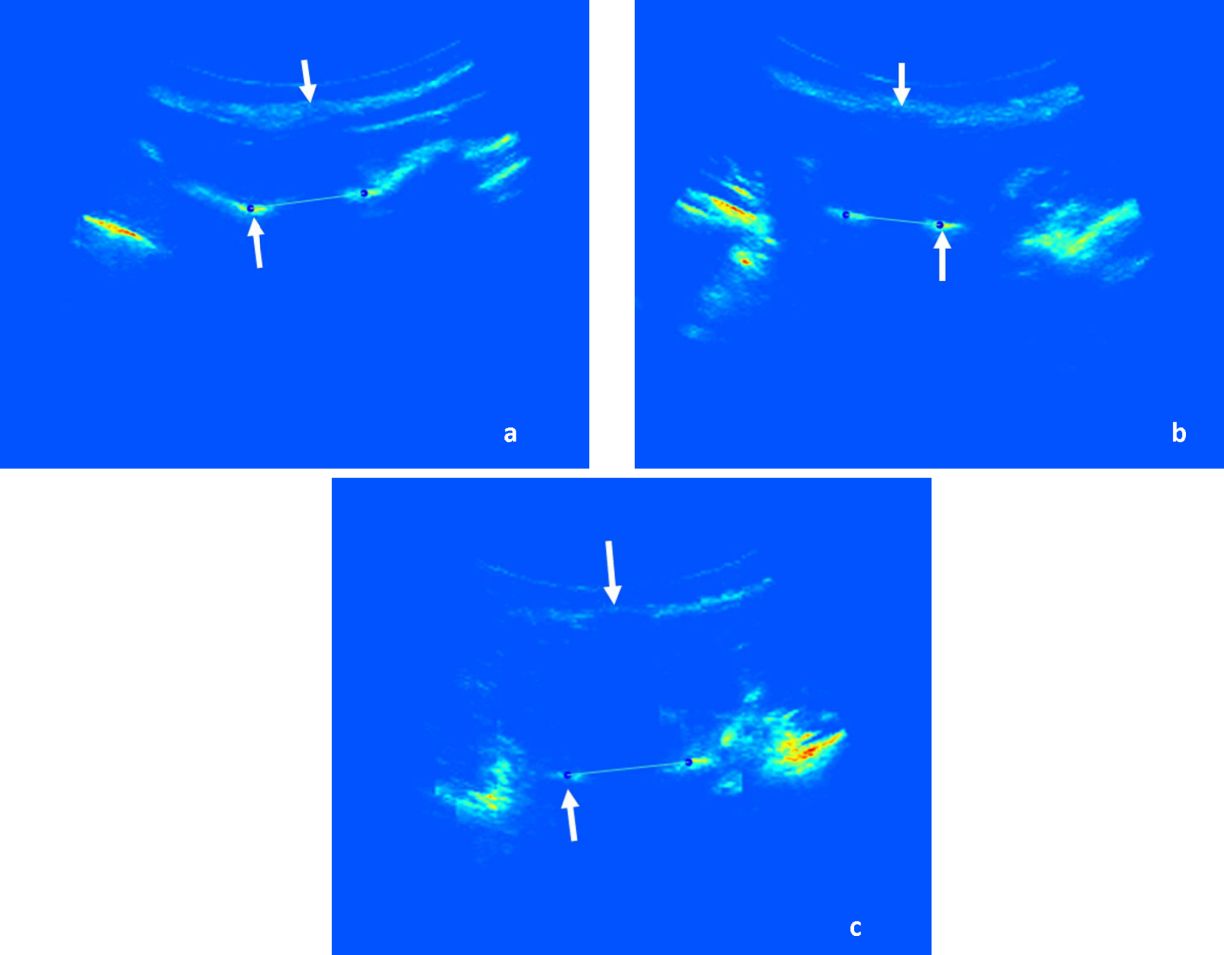
The transverse US images from the same US scan on a 16-year-old girl with BMI 20.5 at different vertebral levels, and the two arrows indicate the distances between skin surface and vertebral lamina, i.e. the muscle thickness covering on the vertebra. (a) T7, (b) T12 and (c) L3.

The attenuation of US signals from soft tissues especially muscles was a major factor influencing the accuracy of US measurements in the lumbar region. Fig 5 showed the US transverse images at the vertebra T7 (a), T12 (b) and L3 (c) from the same US scan on a 16-year-old girl with a BMI of 20.5. The distance between the skin surface and the lamina area (the gap between the two white arrows in Fig 5) shows the thickness of muscle covering the lamina area. When the ultrasound signals penetrate into a subject’s back, the energy exponentially decays with the muscle thickness due to the effect of attenuation and scattering. As a result, less energy reflects back from a thick muscle area. As shown in Fig 5, the muscle thickness in the thoracic area (T7) (Fig 5A) and thoracolumbar area (T12) (Fig 5B) was thinner than in the lumbar area (L3) (Fig 5C). Thus Fig 5C was noisier and had lower intensity than Fig 5A and 5B and was characterized with blurred lamina lines and less image brightness and contrast. The poor image quality decreases the measurement accuracy; therefore, the occurrence of large discrepancy curves appeared more frequently in the lumbar area.

In our study, the measurements also showed minor trend of overestimation (i.e. positive measurement difference) on mild curve and underestimation (i.e. negative measurement difference) on non-mild curves. According to the linear regression equation indicated on Fig 2A, the calculated US measurement is greater than radiographic measurement when Cobb angle<22°, and smaller than radiographic measurements when Cobb angle≥22°. This phenomenon was especially explicit for the large discrepancy curves, i.e. the measurement difference was out of the limits of agreement. As shown in Fig 2B, of all 16 outrange data points, if the radiographic Cobb angles instead of average Cobb are used for evaluation, then all 5 positive points occurs in curves with Cobb < 22° and all 11 negative points occurs in curves with Cobb≥22°. Zheng et al [16] and Brink et al [25] also reported that the relationship between ultrasound Scolioscan angle (x) and the radiographic Cobb angle (y) can be expressed by the equation y = px, where p is a linear coefficient in the range of 1.15–1.20. The ultrasound measurement underestimated the thoracic and lumbar curve severities probably because the posterior landmarks such as spinal process and transverse process were used for the ultrasound method instead of the vertebral bodies used for radiography.

Of all 314 curves from 193 subjects with known weight status, no apparent difference was discovered between the normal and overweight groups, but the small distinction was shown for the underweight subjects. The MAD±SD of the US measurements was only 1.8±1.5° for the underweight group, which was slightly smaller than the normal group (2.2±1.7°) and the overweight group (2.1±1.7°). The reason for this phenomenon was the different reaction of the ultrasound energy to fat and muscles. The density of fat is 20% lower than of muscle [26]. It indicates that there is less energy loss due to tissue absorption when ultrasound beams penetrate the fat layer. On the other hand, the multi-layer structure of back muscle [27] can generate more scattering and attenuation effects and greatly reduce the energy strength, while the fat is a homogeneous matter and an easy path for ultrasound beam propagation. Therefore, the US scans from the underweight subjects with less fat and muscle demonstrate the better image quality and lead to more accurate results while the normal and overweight groups present no distinct measurement difference even though the overweight subjects have more body fat. However due to the limited number of underweight subjects, the effects of body weight still requires more observations. On the other hand, the BMI measurements in this study were only based on height and weight, therefore a good measure of lean body mass to differentiate the influences from muscles or fat are also needed for future study.

**Fig 6:**
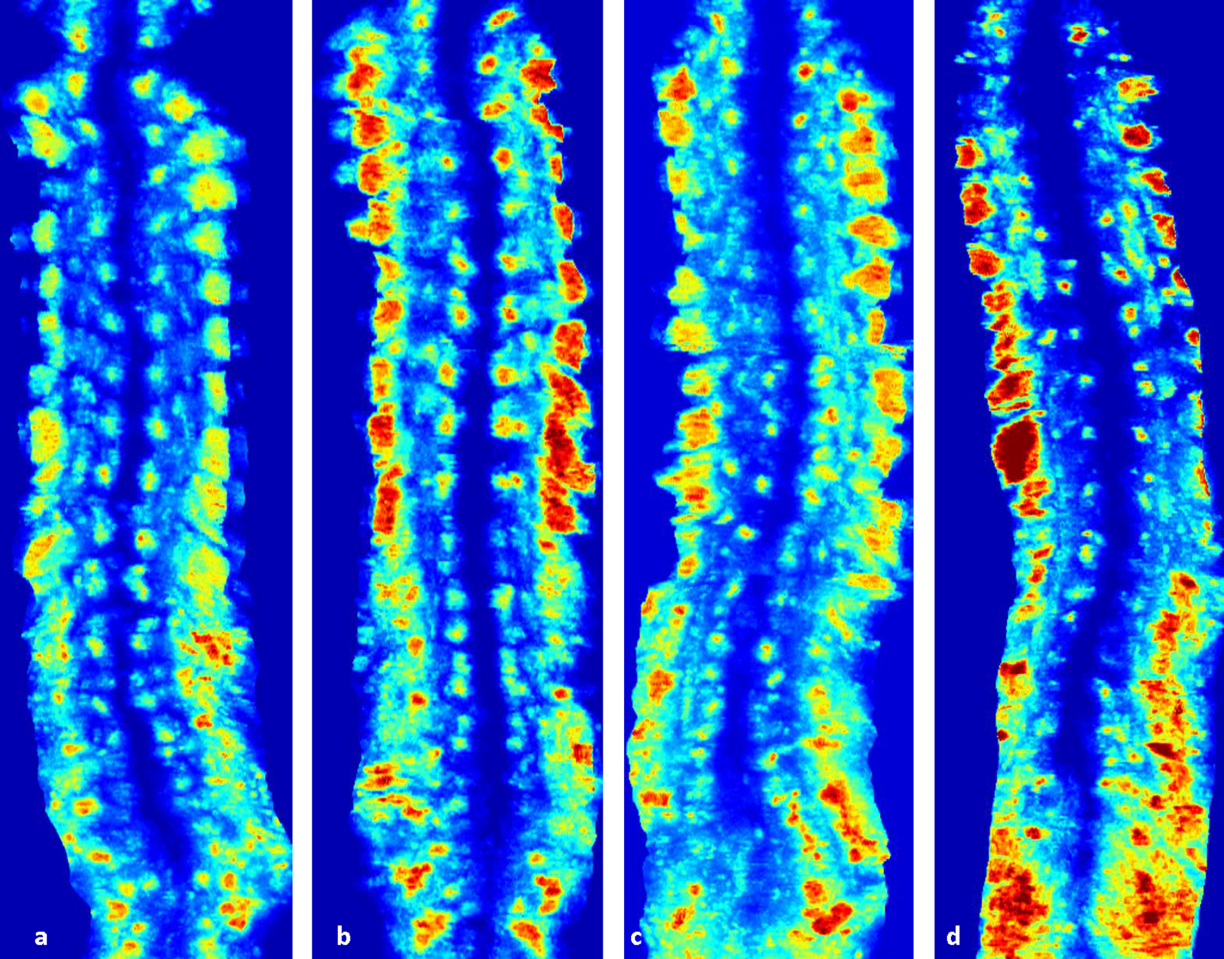
The US coronal images from 4 female subjects scanned by the two operators. (a) Subject A (age:12.1, BMI:17.8, Maximum Cobb angle:12°, Maximum AVR: 1°) scanned by the experienced operator, (b) Subject B (age:12.0, BMI:17.0, Maximum Cobb angle:12°, Maximum AVR: 2°) scanned by a trainee, (c) Subject C (age:15, BMI:24.6, Maximum Cobb angle:26°, Maximum AVR: 11°) scanned by the experienced operator, (b) Subject D (age:16.6, BMI:23.6, Maximum Cobb angle:30°, Maximum AVR: 10°) scanned by a trainee.

Lastly, the different measurement results were noted on the US scans obtained by personnel with different levels of ultrasound operating experience. The scans from the experienced operator showed higher measurement accuracy than the scans from trainee operators, including less large discrepancy curves (3% vs 6%), smaller MAD (1.7±1.5° vs 2.4±1.8°; p<0.001) and higher correlation (0.95 vs 0.90). More skilled operation during US scanning provides better image quality and results in more confidence and consistency of the measurements. Fig 6 showed the US coronal images acquired by the experienced operator vs a trainee operator (Fig 6A vs 6B and 6C vs 6D) from four female subjects (Subjects A to D corresponding to the figure legend). Subjects A and B were the same age with mild Cobb angles, small AVR and BMI, while Subjects C and D were of similar age with moderate Cobb angles, large AVR and BMI. The images from Subjects A and B both clearly indicate all the structures of the vertebrae and spine, but Fig 6C and 6D illustrate very different features. The scan acquired by the trainee (Fig 6D) presented small and unclear lamina pairs and an incomplete image especially in the lumbar area. It indicates the skills and experience of the US operator can affect the US image quality especially for those patients with large curvatures, AVR and BMI.

Ultrasound machine is mobile and low cost. The reliability and accuracy of the Cobb and AVR measurements from ultrasound have been demonstrated by many studies (11–18). Recently, using ultrasound imaging method to identify curve progression in children with idiopathic scoliosis was reported [28]. The curve difference between the proxy Cobb angle measured from the current US image minus the Cobb angle from the previous radiograph was calculated and defined as the threshold value to identify curve progression. The thresholds 4° and 5° presented sensitivities ≥0.90 and specificities ≥0.85 to detect curve progression. Therefore, using US method to follow non-progressive case can reduce more than 70% of the radiographs. Furthermore, ultrasound imaging method has been implemented to assess the spinal curve flexibility on scoliotic surgical candidates, and the result was comparable to the radiographic supine bending method [29]. However, the US technique cannot be applied on subjects who had spinal surgeries. The metal implants inside the body reflect the US strongly and block the US signals to hit the landmarks on the vertebra. Secondly the US imaging method cannot recognize vertebral disc or end plate of vertebrae, wedging of vertebrae cannot be identified. Lastly as indicated in the discussion, the severe AIS with large axial vertebral rotation significantly reduces image quality which can affect accuracy and reliability of the US measurements. On the other aspect, the US imaging method has been applied only to the measurement of coronal curvatures for this stage. The sagittal curvature is a very important topic for our ultrasound spine imaging study in the future. The algorithm of measurement on sagittal curvature is still under development, and more research is required to validate the method for the present.

## Conclusions

Only 4% missing curves and 5% large discrepancy curves were demonstrated for the US measurement in comparison with the results from conventional radiography. The two main factors causing the missing curves were small severity of the curves and upper spine locations. There is a higher chance of the large discrepancy curves being in the main thoracic and lumbar regions. A large axial vertebral rotation in combination with a Cobb angle ≥25° can influence the measurement accuracy and result in large differences between the US and radiographic measurements. US measurements show no apparent distinction between the normal and overweight subjects. A more skilled scan operator can improve the US image quality and lead to more accurate measurements especially for those subjects with larger curvatures, AVR and BMI.

## Supporting information

S1 Data Original data file of the 200 subjects. (XLSX)
